# Case of Traumatic Tetanus—Recovery

**Published:** 1877-10-20

**Authors:** G. A. Evans


### CASE OF TRAUMATIC TETANUS —RECOVERY.

READ BEFORE MEDICAL SOCIETY, COUNTY KINGS, N. Y., BY G. A. EVANS, M.D.

Mr. Jenkins, aged thirty-five, born in England, married, machinist by occupation, well developed, and has always enjoyed go?d health.

On Aug. 7th, 1876, while adjusting some heavy machinery, he accidently received a deep puncture in the outer side of the middle third of his right leg, from a shaft of steel which tapered abruptly and irregularly to a point. Its extraction by a fellow-workman was followed by a protrusicn of fat and muscle, together with profuse hemorrhage.

Aug. 23d: Patient consulted me about pain in his epigastrium, stiffness of the muscles of mastication and deglutition, and general lassitude. I ordered him to take two scruples of the bromide of potassium every hour for eight doses, and applied about twenty small blisters along his spine with cantharidal collo–dion.

Aug. 26th: Called to see patient, accompanied by Dr. J. H. Hunt. The wound was in good granulating condition, general muscular rigidity had permanently established itself, clonic spasms occasionally occur, the pharyngeal and respiratory muscles are but slightly affected, intellect clear, patient rational and very cheerful. I ordered him to take twenty two minim doses of the tincture of cannabis indica every hour (equal to one grain of the purified extract.)

Aug. 27th: No change in his condition.

Aug. 28th: Patient the same; dose ot cannabis indica increased to thirty minims.

Aug. 29th: Patient's condition remains unchanged. Up to this date he was been able to get an occasional nap. The appetite has remained good, notthstanding his inability to take other an liquid food, and through a tube. He knows the gravity of his situation, but feels confident of recovery. I discontinued giving him cannibas indica, and decided to try the nitrite of amyl, which I ordered to be taken on sugar in five minim doses every three hours until my return.

Aug. 30th: I was informed that "after he had taken the first dose, clonic spasms, which, up to this time, had occasioned comparatively no discomfort, developed to an alarming extent." Tonic rigidity has become more marked, the characteristic sardonic expression has appeared to a noticeable degree, the ability to sleep has disappeared, and his previous good appetite has given way to a distressing nausea. Considering this affection among the opprobria of medical art, I determined to give the nitrite of amyl still further trial. Accordingly, I ordered ten minims to be given every third hour.

I saw the patient again in the evening, and finding him rapidly sinking, I gave an experimental dose of twenty minims of the nitrite of amyl on sugar, as a last trial, for the exposure of its much-vaunted efficacy in this affection, and watched for the result.

As far as this case was concerned, the drug not only proved itself useless to benefit, but demonstrated its ability to hasten a fatal termination by its increasing tonic and clonic contractions, both in force and frequency. Opisthotonos disappeared, only to give place to emprosthotonos. So violent were the paroxysms, that it was impossible to confine him to any given part of the room. Vomiting and the involuntary passage of fæcal matter contributed to his general torment; spasmodic respiration occasioned a marked degree of cyanosis of the face and neck. The relation between cause and effect was so apparent to the relatives, that they protested against my further using the drug. I abandoned its further administration, convinced that I had taken away my patient's chances (slight as I considered them) of recovery. The tincture cannabis indica was ordered to be given him in thirty minim doses every hour

Aug. 31st: Patient improved in every respect. He has had seven hours' unbroken sleep, the appetite has returned, opisthotonos and emprosthotonos have almost entirely disappeared, clonic spasms still persist, but are less in force and frequency. Tincture cannabis indica, forty minims every hour.

Sept. 1st: Improvement continues.

Sept. 2d: Tetanic spasms increasing in force and frequency, opisthotonos again developing, appetite disappearing; patient has had no sleep since yesterday. An examination of the bottle in which the medicine was kept revealed "that the cannabis indica had precipitated on its sides." A fresh supply was ordered.

Sept. 3d: Patient still continues to grow worse. Every muscle seems to be affected with clonic spasms; general tonic rigidity has endured since the beginning, but does not disturb him except in mastication, deglutition and respiration; he has had no sleep since day before yesterday, and has eaten nothing. Another examination of the medicine proved that the cannabis indica has again precipitated from the syrup with which it had been prescribed. The pure tincture was ordered to be given in forty minim doses every hour.

Sept. 3d, evening: Appetite has returned, patient takes food ravenously; he has had two hours' sleep and is quite cheerful; otherwise his condition is much the same as it was this morning. Dose of tincture cannabis indica increased to sixty-six minims every hour.

Sept. 4th: Appetite good, patient cheerful; has had three hours' sleep; clonic spasms still continue, but not so severe. Tincture cannabis indica, eighty-eight minims every hour.

Sept. 4th, evening: Dr. H. F. Williams called with me. Clonic spasms much less in force than they were this morning, appetite good, patient cheerful; has had four hours' sleep. Tincture cannabis indica, ninety minims every hour.

Sept. 5th: Contractions, tonic and clonic, much milder, appetite ravenous; slept six hours. Tincture cannabis indica, one hundred minims every hour.

Sept. 6th: Improvement continues. The muscles of respiration cease to be affected. Patient is able to move his legs without provoking spasms. Tincture cannabis indica, one hundred and twenty-two minims every hour.

Sept. 7th: Improved in every respect. Patient is acting very silly this morning, and is inclined to be witty at his own expense. Tincture cannabis indica was ordered to be taken in one hundred and thirty-two minim doses every hour (equal to 6 grs. of purified solid extract), in order to produce, if possible, that dreamy ecstasy so often described in the text-books.

Sept. 8th: Patient complains of nothing except the inability to satisfy his appetite. His principal occupation since I saw him yesterday has been to tell silly and extravagant stories about himself. Tonic rigidity is present to a slight extent only, clonic spasms have disappeared. Tincture cannabis indica, sixty minims every hour.

Sept. 9th: During the night patient experienced some severe clonic spasms of the muscles of the back. The nurse on her own responsibility increased the dose of the tincture to ninety minims, after which the spasms ceased to occur.

Sept. 11th: Patient sitting up. The wound has entirely healed by granulation. The tincture cannabis indica was given in ninety minim doses for a few days, when it was discontinued altogether.

